# Enhancing tomato drought resilience with organic amendments and local landraces

**DOI:** 10.1038/s41598-025-12098-0

**Published:** 2025-07-18

**Authors:** Yüksel Tüzel, Hüseyin Hüsnü Kayıkçıoğlu, Tunç Durdu, Omar Saley Harouna, Ulaş Tunalı, Gölgen Bahar Öztekin, Abdulletif Tutal, Mahmut Tepecik, Tansel Kaygısız, Bisem Nisa Kandemir, Nazim S. Gruda

**Affiliations:** 1 Faculty of Agriculture, Department of Horticulture, Ege University, 35100 Bornova-İzmir, Türkiye; 2 Faculty of Agriculture, Department of Soil Science and Plant Nutrition, Ege University, 35100 Bornova-İzmir, Türkiye; 3 Teknotar Ltd. Co, 35070 Bornova-İzmir, Türkiye; 4 Agrodrip Irrigation Systems, 35570 Kemalpaşa-İzmir, Türkiye; 5 Ege University Graduate School of Natural and Applied Science, 35100 Bornova-İzmir, Türkiye; 6 Faculty of Agriculture, Department of Horticulture, Ege University, 35100 Bornova-Izmir, Türkiye; 7 Department of Horticultural Sciences, University of Bonn, INRES – Institute of Crop Science and Resource Conservation, 53113 Bonn, Germany

**Keywords:** Vermicompost, Biochar, Yield, Water use efficiency, Soil biota, Additive main effects and multiplicative interaction, Plant sciences, Plant stress responses, Abiotic, Drought

## Abstract

Climate change has continuously increased drought risk. Alternative economic, sustainable, and practical solutions are still needed to address this risk. In this study, conducted in Izmir, Türkiye, we used organic amendments, biochar, and vermicompost in combination with local tomato landraces as a strategy against drought stress. Four landraces, ‘Olympia,’ ‘Areti,’ ‘TR40430’ and ‘TR43513’ were selected from the Mediterranean Basin according to their performance to drought within the scope of the VEGADAPT Prima Project. ‘Moneymaker’ was used as a control under three irrigation treatments. Full irrigation (Ir100) was carried out to replenish the soil moisture to field capacity. For deficit irrigation, 70% (Ir70) and 40% (Ir40) of the water applied in the Ir100 irrigation was provided, respectively. Utilization of stable carbon-rich organic amendments mitigated the adverse effects of limited irrigation on plant growth, yield, water use efficiency, and soil microbial activity. Plant height and fresh and dry weight of vegetative parts were decreased, while leaf thickness and chlorophyll index were increased in the deficit irrigation. The total yield decreased by over 45% in Ir40 compared to Ir100 and Ir70. The total soluble solids, titratable acidity, fruit dry weight, and fruit firmness increased with water deficit, while fruit juice pH decreased. Irrigating at Ir70 levels with vermicompost or biochar significantly enhanced microbial activity, maintaining or improving function under the stricter Ir40 regime, though with different effects. The additive main effects and multiplicative interaction (AMMI) model was used to evaluate the overall adaptability of the studied landraces. ‘TR40430’ gave the best total yield on average, however, it showed the lowest results in terms of water use efficiency (WUE). ‘Areti’ was as good as cv. ‘Moneymaker’ in WUE. AMMI analysis also indicated the improvement of the effects of microbial activity on landraces. These results highlight the crucial role of organic amendments in enhancing soil resilience to water scarcity and supporting sustainable agricultural practices amid climate challenges.

**Supplementary Information:**

The online version contains supplementary material available at 10.1038/s41598-025-12098-0.

## Introduction

Human activities such as using fossil fuels, industrialization, destroying forests, and increasing the world population increase the greenhouse gas balance^1,2^. During the last two decades, the total warming effect from greenhouse gases due to human activities has increased by 45% ^3^, making greenhouse gas emissions the most significant contributor to climate change^4^. Climate change increases drought risk, mainly due to temperature and precipitation changes. According to future projections, the drought frequency and severity are expected to impact the Mediterranean Region significantly^5^. Türkiye has faced significant changes in its climate. Temperatures have been consistently rising during the last three decades, and it is projected to increase by ~ 2.5 °C until the 2060s and over 5 °C by the end of this century^6^.

Natural drought types can be “climate-induced” or “human-induced”^7^. Based on the severity and duration, drought causes environmental, social, and economic impacts. The impact on agriculture and public water supply predominates for most regions^8^. Drought further accelerates soil salinization and intensifies plant damage, adversely affecting yield and quality, even in protected cultivation^2^. For instance, yield is influenced by up to 50% in many horticultural crops^9^.

Tomato is one of the most widely cultivated and economically important vegetable crops worldwide. It has become a key crop in the Mediterranean Region’s open-field and protected cultivation systems^10^. Tomato exhibits moderate tolerance to salinity, whereas it is highly susceptible to severe damage under drought stress^11^. Tomato is used in abiotic stress research due to its broad cultivation range, nutritional importance, and moderate salinity sensitivity^12^. However, the genus *Solanum* has extensive genetic variation, particularly among tomato landraces and wild relatives^10,13–15^. Given its global distribution, nutritional value, and vulnerability to environmental stressors, enhancing tomato resilience to climate-induced water limitations is a critical priority for sustainable agriculture, particularly in semi-arid regions.

Different strategies in agriculture could be adopted^16–19^ for abiotic stress tolerance in tomato production. To mitigate the effects of drought, deficit irrigation is one of the strategies to reduce water use, particularly in arid and semi-arid regions^20–22^. The effects of drought can be cultivar-specific^23^ highlighting the importance of biodiversity as a vital tool for enhancing resilience to drought^24^. Several studies have documented genetic variability among local landraces^10^ and cultivars^25,26^ and their responses to drought^27–30^.

Additionally, using organic amendments can also contribute to the mitigation of drought stress by enhancing soil microbial activity^31–35^ improving soil physical and chemical properties^36–38 ^increasing water use efficiency^39,40^ and/or decreasing reactive oxygen species^41,42^. However, the response may be affected by the type of organic amendment^43^ and/or the use of beneficial microorganisms^44^.

Biochar is charcoal produced through biomass pyrolysis of biomass such as agricultural residues, manure, waste, or wood products. Highly porous and large surface area make it an effective soil amendment and carbon sequestration tool. Biochar improves growth and yield by improving soil fertility, water, and nutrient retention under abiotic stresses^43,45^. Under water stress/deficit conditions, biochar is found to be a sustainable tool in tomato production due to its effects on yield and soil physical properties^46,47^.

Vermicompost, also known as worm compost or worm castings, is a nutrient-rich organic fertilizer and soil amendment produced by earthworms’ decomposition of organic matter. The multifaceted benefits of vermicompost make it a valuable tool for promoting soil health, enhancing plant growth, and supporting sustainable agricultural practices. The application of vermicompost increased plant growth and yield in different crops, such as lettuce^48^, canola^49^, eggplant^50^ and tomato^51^. Vermicompost is also used as a biostimulant in soilless culture systems to enhance the resilience to abiotic stresses and improve vegetable growth, yield, and produce quality^52,53^.

Biochar and vermicompost have been tested in different vegetables to determine their effects on growth, yield, physiological traits, and/or produce quality. However, no existing studies evaluated the interactive effects of local tomato landraces, organic amendments, and deficit irrigation strategies on plant performance and soil microbial functioning under greenhouse conditions in the Mediterranean Region. Therefore, our study presents a novel and integrated framework that simultaneously considers genotype (landraces), management (organic amendments), and environmental stress (drought) with physiological, agronomic, and microbial responses as a composite assessment of drought resilience. It addresses a key knowledge gap at the intersection of soil management, plant genetic diversity, and water scarcity, offering science-based insights into the development of resilient and resource-efficient tomato production systems compared to conventional practices, and could fill a meaningful research gap in sustainable crop management under climate stress conditions in arid and semi-arid regions.

Hence, we hypothesize that (1) the application of organic amendments will differentially affect soil microbial activity -measured through key enzymatic indicators- under varying levels of water deficit (Ir70 and Ir40), depending on amendment type with vermicompost and/or biochar being more effective in sustaining microbial activity under moderate and/or severe irrigation deficiency, and (2) organic amendments, in combination with irrigation regimes, will lead to distinct responses in vegetative growth, yield components, and fruit quality across different tomato landraces. Specifically, organic soil amendments could mitigate adverse effects of water deficit on plant and soil functions in tomato cultivation we aimed to investigate how organic materials such as biochar and vermicompost influence plant growth, yield, fruit quality, irrigation water use efficiency, and soil microbial activities in tolerant landraces sourced from various regions of the Mediterranean Basin. This research seeks to fill existing knowledge gaps and contribute to more sustainable agricultural practices in the area, ultimately promoting enhanced resilience against climate variability and offering a viable strategy to mitigate the adverse effects of water scarcity on tomato production in Mediterranean climates.

## Materials and methods

This research was conducted in a polyethylene-covered greenhouse (12 × 44 m) during the spring season of 2021 at the Department of Horticulture of Ege University Faculty of Agriculture (Bornova-İzmir, Türkiye, 38°27’17.03’’N, 27°14’17.71’’E).

### Plant material

**Fig. 1:**
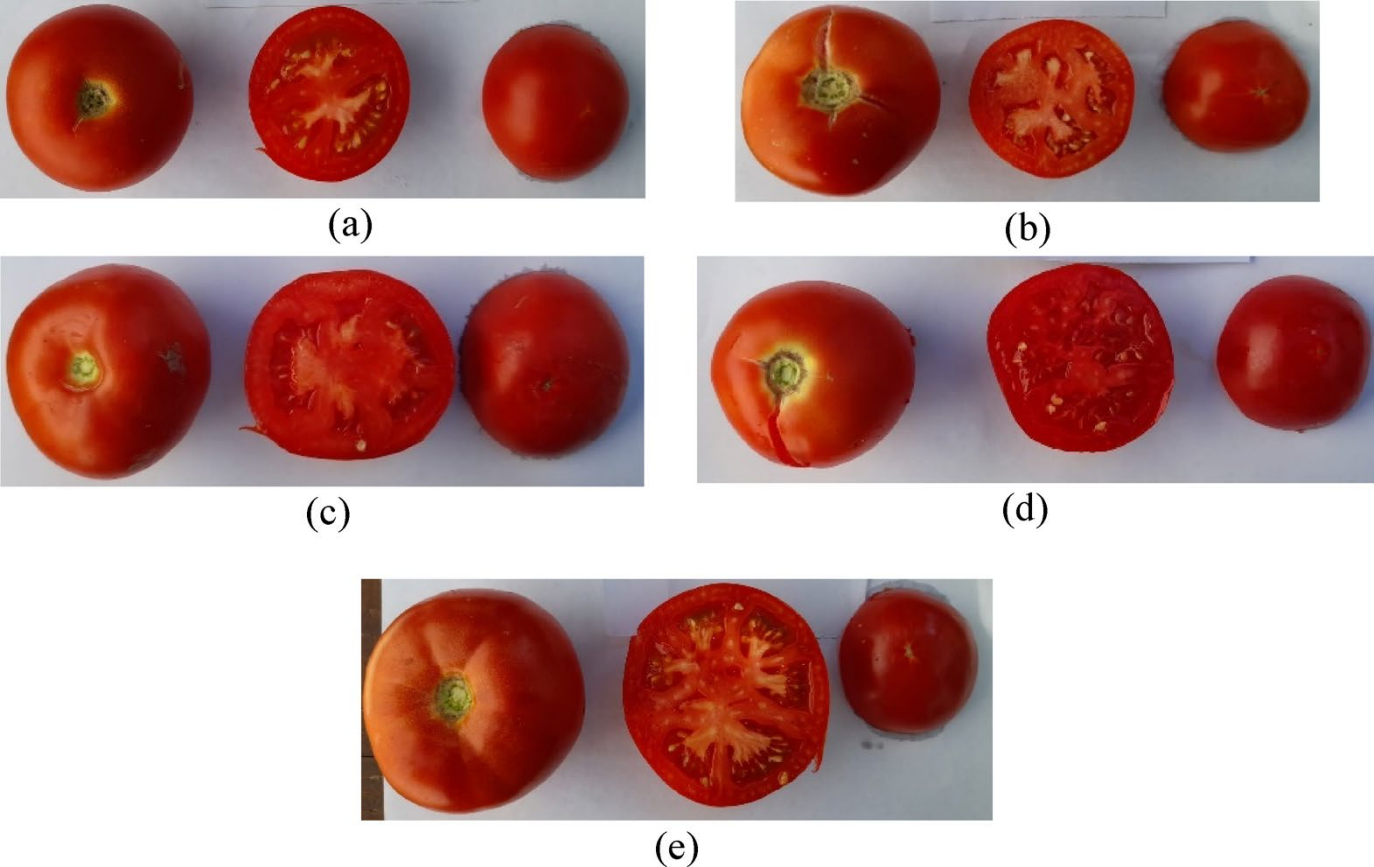
(**a**) ‘Moneymaker’; (**b**) ‘Olympia’; (**c**) ‘Areti’; (**d**) ‘TR40430’; (**e**) ‘TR 43513’.

Within the Prima VEG-ADAPT Project (Adapting Mediterranean Vegetable Crops to Climate Change-Induced Multiple Stress) scope, tomato landraces from different parts of the Mediterranean Region were tested against various abiotic stresses. Among the ones having better performance based on the ranking under drought stress, five tomato landraces, including control (cv. ‘Moneymaker’), were used in this study (Table 1; Fig. 1).

**Table 1:** Origin and some properties of the used tomato accessions/varieties.

No	Landraces	Institute / Country	Growth
1	cv. ‘Moneymaker’	INRAE / France	Indeterminate
2	‘Olympia’	AUA / Greece	Semi-determinate
3	‘Areti’	AUA / Greece	Semi-determinate
4	‘TR40430’	ETAE / Türkiye	Determinate
5	‘TR43513’	ETAE / Türkiye	Indeterminate

INRAE: Institut National de la Récherche Agronomique - Génétique et Amélioration des Fruits et Légumes, AUA: Agricultural University of Athens, ETAE: Aegean Agricultural Research Institute.

### Seedling growing

Before sowing, the seeds were disinfected by soaking in a solution of 2.5% HCl (hydrochloric acid) in a sieve for 1 min, then rinsed under tap water and subsequently soaked in pure water for 1 min. The seeds were dried before use. The peat was thoroughly mixed and moistened, then filled into 128-cell disinfected styrofoam trays. For each landrace, 128 seeds were sown in each tray on February 22, 2021. After seed sowing, trays were placed in a germination chamber with a temperature of 22 °C (night and day) and 85% humidity for three days. Afterward, trays were transferred to the nursery until planting. During this period, the seedlings were fertigated (20-20-20 + TE, Agroleaf Power, Altıntar, Bornova/Türkiye) using a boom irrigation system.

### Organic soil amendments

Two organic materials, vermicompost (VC) (worm castings) and biochar (BIO), were used as soil amendments. Vermicompost (Çamlı Yem Besicilik, İzmir/Türkiye) was obtained by processing *Eisenia foetida* worms over one year using the vermiculture method, starting from cattle manure. Biochar (Bilen Kömür Enerji Gübre San. ve Tic. Ltd. Şti., Mersin/Türkiye), on the other hand, was produced through slow pyrolysis at 550 °C using by-products of a 3-phase olive cake. Some properties of biochar, vermicompost, and soil are given in Table 2.

**Table 2:** Some physical and chemical properties of organic amendments and the soil.

Parameters	Biochar	Vermicompost	Parameters	Soil
pH	6.71	6.92	pH	8.24
Total salt (µS cm^− 1^)	2.16	5.56	Total salt (µS cm^− 1^)	925
Total N (%)	3.78	2.61	Lime (%)	1.32
C (%)	31.09	32.52	Sand (%)	60.72
C/N	8.23	12.44	Silt (%)	25.28
Organic matter (%)	53.60	56.07	Clay (%)	14.00
Humidity (%)	5.50	19.11	Texture	Sandy loam
Total K (%)	0.15	1.28	Organic matter (%)	1.78
Total Ca (%)	3.02	3.99	Total N (%)	0.062
Total P (%)	2.12	1.62	Available P (mg kg^− 1^)	21.8
Total Mg (%)	0.55	0.67	Available K (mg kg^− 1^)	216.5
Total Na (mg kg^− 1^)	2538.40	1713.83	Available Ca (mg kg^− 1^)	4162.2
Total Cu (mg kg^− 1^)	114.48	22.51	Available Mg (mg kg^− 1^)	597.7
Total Fe (mg kg^− 1^)	1763.08	2645.56	Available Na (mg kg^− 1^)	107.4
Total Mn (mg kg^− 1^)	73.30	141.26	Available Fe (mg kg^− 1^)	0.88
Total Zn (mg kg^− 1^)	531.04	162.10	Available Zn (mg kg^− 1^)	1.93
Total B (mg kg^− 1^)	34.78	71.24	Available Cu (mg kg^− 1^)	4.81
			Available Mn (mg kg^− 1^)	4.41

### Planting and cultivation practices

**Fig. 2:**
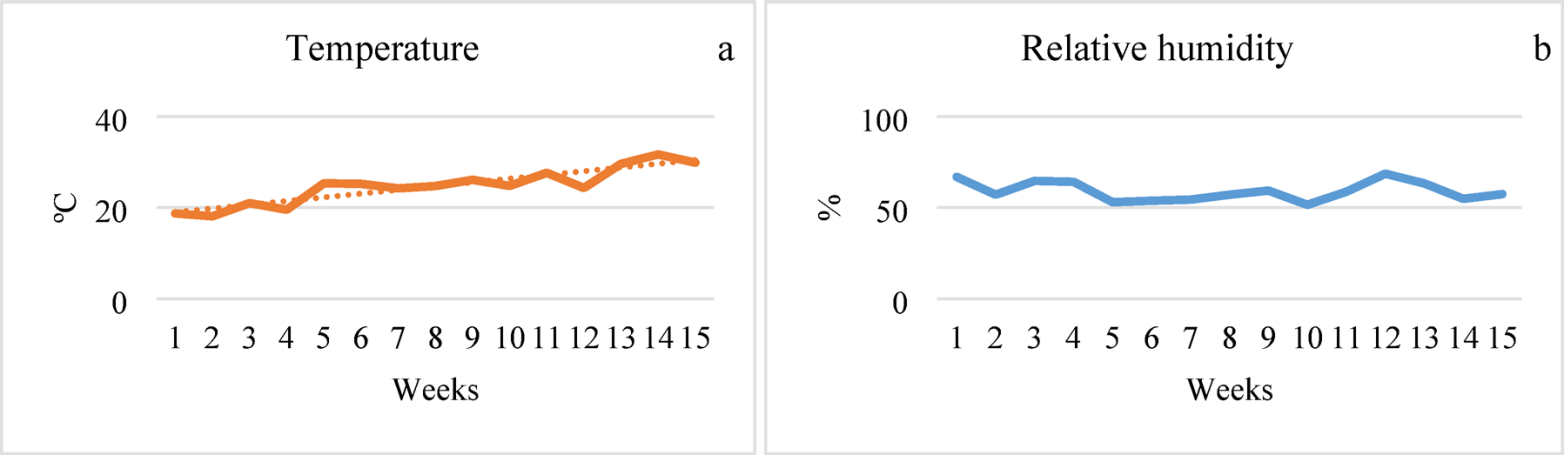
Weekly mean temperature (**a**) and relative humidity (**b**) in the greenhouse.

The soil of the greenhouse was tilled before planting. Fertilization was applied according to^54^ recommendations at rates of 200 kg N ha^− 1^, 100 kg P_2_O_5_ ha^− 1^ and 600 kg K_2_O ha^− 1^. Vermicompost and biochar were applied to each plot at a rate of 1 kg m^− 2^ before planting. Seedlings were grown in double rows with row spacing of 80 × 40 × 50 cm and 3.5 plants m^− 2^ on March 29, 2021. Each plot also included unplanted rows and border rows. Cultivation practices such as pruning, pollination, pest and disease control, and harvest were conducted according to Öztekin and Tüzel^55^. During the production, some pests and diseases such as red spider mites, whiteflies, and powdery mildew were observed, and pesticides were applied uniformly to all treatments. Plant cultivation ended on July 10, 2021. The mean temperatures were 19.9, 25.2, 27.2, and 30.4 ºC in April, May, June, and July, while the average relative humidity was 58.4% (Fig. 2).

### Treatments

We used a split-plot design with randomized blocks, two field-level replications, and six plants in each treatment. In the main plots, cultivation was carried out under three irrigation conditions: Full Irrigation (Ir100) and two different water deficits (Deficit1: Ir70 and Deficit2: Ir40). To mitigate the impact of drought stress on the plots, (1) Vermicompost-VC, (2) Biochar-BIO, and (3) Control-CONT treatments were implemented with five landraces, namely cv. ‘Moneymaker’, ‘Olympia’, ‘Areti’, ‘TR40430’, and ‘TR43513’. “Unplanted” rows were included in each plot to compare the changes in microbial communities with planted ones.

### Irrigation system

A drip irrigation system was used, and irrigation water collected in the main tank was passed through a filter and transported into the greenhouse through 25 mm PVC main pipes. After adjusting the pressure with a pressure regulator, the water was passed through calibrated watermeters. It was then distributed to the plots using 20 mm pipes and reached the plants through 16 mm lateral pipes. For full irrigation (Ir100), the plant root zone was allowed to consume 20% of the available water holding capacity, and irrigation was carried out to bring the soil moisture level back to field capacity. For deficit irrigation treatments, 70% (Ir70) and 40% (Ir40) of the water applied in the complete irrigation treatment was provided, respectively.

Soil moisture and electrical conductivity were monitored using soil moisture sensors (Teros 12 Moisture / Temperature / EC and GS3) placed at depths of 15 and 45 cm. The data was collected hourly and recorded with data loggers (Degacon-EM50). The sensors were calibrated for the existing soil characteristics before the experiment. Therefore, irrigation treatments were controlled using 3 data loggers and 13 moisture sensors placed in the middle section of the experiment’s plots. The amount of irrigation water applied to each treatment was determined by multiplying the decrease in moisture content at a root depth of 0–60 cm, as determined by the moisture sensor measurements, with the irrigated area, wetting percentage, and the coefficient related to that specific treatment.

### Measurements and analysis

The length from the hypocotyl to the growth tip of three plants per repetition was measured on-site at the end of the growing season with a meter tape to determine plant height (m). Leaf thickness (mm) was measured on ten leaves per replicate using a micrometer (Mitutoyo IP65, Kawasaki, Japan) on the third leaf of ten plants downward from the growing tip. Root and vegetative shoot (leaf + stem) parts of four plants per replicate were harvested at the end of the growing cycle and weighed without any delay for fresh weights (FW, kg plant^− 1^). After drying in an oven at 65 °C till reaching a constant weight, dry weights (DW) of the vegetative parts and roots (kg plant^− 1^) were measured using a precision scale.

Fruits collected from 6 plants in each repetition were individually weighed immediately at each harvest, and yield values of each treatment were summed to calculate the total yield (kg m^− 2^). The number of fruits collected at harvests was counted, and the total fruit number (number m^− 2^) for each treatment was determined at the end of the study. Average fruit weight (g fruit^− 1^) was calculated by dividing the total yield by the total number of fruit.

Quality analyses were performed on tomatoes as three replicates with 10 fruits each, specifically on fully ripened 3rd -4th truss fruit samples collected on July 05, 2021. The harvest stage was decided according to the tomato color scale, numbers 11 and 12 (CTIFL, Tomato Code Colour, Paris, France). Tomato samples harvested in the early morning were transferred from the greenhouse to the laboratory without delay. Juice extracted from 3 to 4 fruits using a blender was strained through a cloth, and a few drops of the filtrate were measured with a digital handheld refractometer (Euromex RD 645, Arnhem, The Netherlands) to determine soluble solids content (%). Juice pH was measured using a benchtop pH meter (Mettler Toledo SevenEasy, Giessen, Germany) by immersing the pH probe into the juice. Firmness was immediately measured using a hand-type penetrometer (FT 011, Effegi, Alfonsine, Italy), using the standard 7.94 mm diameter probe on the skin of five randomly selected fruits from each treatment and expressed as Newton. The sample was titrated with a 0.1 N NaOH solution until a pH of 8.01 was reached. Titration was used to calculate titratable acidity (TA, mval 100 mL^− 1^) based on the amount of NaOH used^56^. Vitamin C content (mg 100 g^− 1^), total phenolic content (mg gallic acid equivalent 100 g^− 1^ fresh weight), and antioxidant activity (µM trolox equivalent g^− 1^ fresh weight) of tomato fruits were determined spectrophotometrically according to Pearson^57^Thaipong et al.^58^and Swain and Hillis^59^respectively. The Folin-Ciocalteu calorimetric method was modified to determine total phenolic content, and the Ferric Reducing Antioxidant Power (FRAP) method was used to assess antioxidant activity^60^.

The irrigation water applied to the plants was measured with water meters near the plots. The applied irrigation water amount (mm and L plant^− 1^) was calculated, and water use efficiency (WUE) was determined by dividing the plant water consumption by the total yield, following Zotarelli et al.^61^. Changes in available soil moisture capacity throughout the production period were also provided (%).

To predict changes in soil biota, soil samples were taken from a 0–15 cm depth on April 8, 2021, 15 days after applying organic soil amendments. The second set of soil samples was taken on 16 July 2021, 114 days after the treatment at the end of the growing cycle. All soil microbial and enzymatic assays were performed using three biological replicates per treatment, based on separately collected subsamples from each plot. They were immediately stored at 4 °C in plastic bags until microbiological and enzymatic activities were assayed. Microbial activities of fresh soils were determined within 30 days of sample collection. Basal soil respiration (BSR)^62,63^, potential N-mineralization (N_min_)^64^, alkaline phosphatase activity (ALKPA, EC 3.1.3.1)^65,66^, dehydrogenase activity (DHG, EC 1.1)^67^, β-glucosidase activity (GLU, EC 3.2.1.21)^68^ and urease enzyme activity (UA, EC 3.5.1.5)^69^ were measured in the soil samples. Results were presented as the average of the two sampling periods, and plant presence (No Plant and Plant) was added to the main factors of irrigation and organic soil amendments.

### Statistical analysis

A three-factor experiment (irrigation, landraces, and organic soil amendments) was conducted using a split-plot randomized block design with two field-level replications due to the number of treatments and space availability in the greenhouse and statistically analyzed to evaluate yield, water use efficiency, plant growth, and fruit quality. Data were subjected to variance analysis to determine any statistically significant differences among variables using the SPSS statistical analysis software package (version 25.0). Duncan’s test was conducted to identify the differences between the means. According to the F-test, the significance levels ns, *, and ** indicate statistical insignificance, not significant (*P* > 0.05), 0.01 < *P* ≤ 0.05, and *P* ≤ 0.001, respectively. Standard error bars are provided on the bars in the graphs.

In addition, a combined analysis of variance (ANOVA) was performed to evaluate the presence of genotype-environment interaction (GEI) on total yield and WUE variables using R statistical software version 4.2.2. To apply additive main effects and multiplicative interaction (AMMI) and biplot modeling, irrigation and soil amendment interactions were considered separate and autonomous environments and analyzed using the metan package^70^. AMMI analysis was preferred to provide a scientific basis for landraces’ overall performance and their responses to environmental variability^71^. The performance of five landraces in nine environments (three organic soil amendments x three irrigations) was subjected to the AMMI analysis based on the following model^72^:

$$\:{\mu\:}_{ij\:}=\:\mu\:+{\alpha\:}_{i}+{\tau\:}_{j}+{{\Sigma\:}}_{k=1}^{p}{\lambda\:}_{k}{\alpha\:}_{ik}{t}_{jk}+{\rho\:}_{ij}+{\epsilon\:}_{ij}$$

where α_i_ is the additive effects of genotype, τ_j_ is the environmental mean, λ_k_ is the singular value for the *k*th interaction principal component axis (IPCA), t_jk_ is the *j*th element of the *k*th eigenvector, and α_ik_ is the ith element of the kth eigenvector.

The biplot analysis categorized the interactions between irrigation and soil amendments as environment, while landraces were labeled separately (Table 3).

**Table 3:** Labeled irrigation and soil amendments interactions and landraces.

Environment (Irrigation x Organic Soil Amendments)		Landraces	
Label	Subject	Label	Landrace
E1	Ir100 + Cont	G1	‘Moneymaker’
E2	Ir100 + Ver	G2	‘Olympia’
E3	Ir100 + Bio	G3	‘Areti’
E4	Ir70 + Cont	G4	‘TR40430’
E5	Ir70 + Ver	G5	‘TR43513’
E6	Ir70 + Bio		
E7	Ir40 + Cont		
E8	Ir40 + Ver		
E9	Ir40 + Bio		

## Results

### Yield

**Fig. 3:**
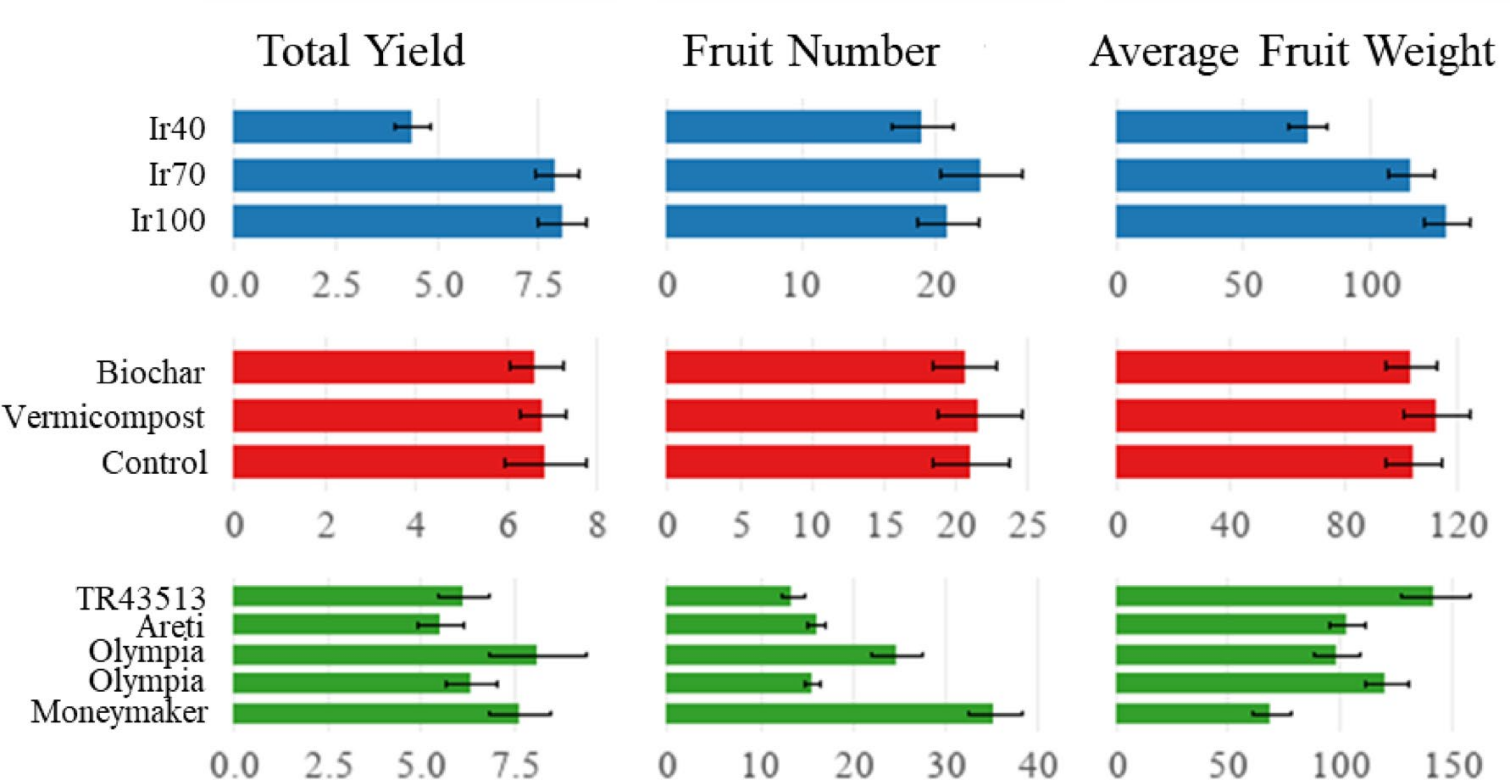
Main effects of irrigation, landraces, and organic amendments on yield parameters (Error bars show standard errors).

The main effects of treatments, excluding organic soil amendments and interactions, were significant (Table 4). Irrigation affected yield parameters: total yield, fruit number, and average fruit weight. It was determined that the highest values were obtained from Ir100 and Ir70. In Ir40, the total yield decreased by 46.0%, the fruit number decreased by 8.7%, and the fruit weight decreased by 42.1%. Landrace response was significantly different, and the highest total yield was obtained from ‘Areti’, followed by ‘Moneymaker’ and ‘Olympia’. The highest total fruit number per unit area was in ‘Moneymaker’, while the average fruit weight was obtained from the ‘TR43513’ (Fig. 3).

**Table 4:** Main and interaction effects of irrigation, landraces, and organic amendments on some yield parameters.

Independent Variables	Total Yield	Fruit Number	Average Fruit Weight
Irrigation (A)	**	*	**
Landrace (B)	**	**	**
Org. Amendment (C)	*ns*	*ns*	*ns*
AxB	**	**	**
AxC	**	**	**
BxC	**	**	**
AxBxC	**	**	**

**: *P* < 0.01, *: *P* < 0.05, ns: not significant.

**Fig. 4:**
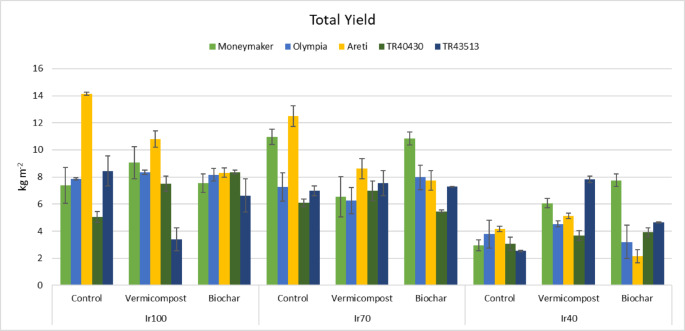
Interaction effects (*P*: 0.002) of irrigation, landraces, and organic amendments on total yield (Error bars show standard errors).

**Fig. 5:**
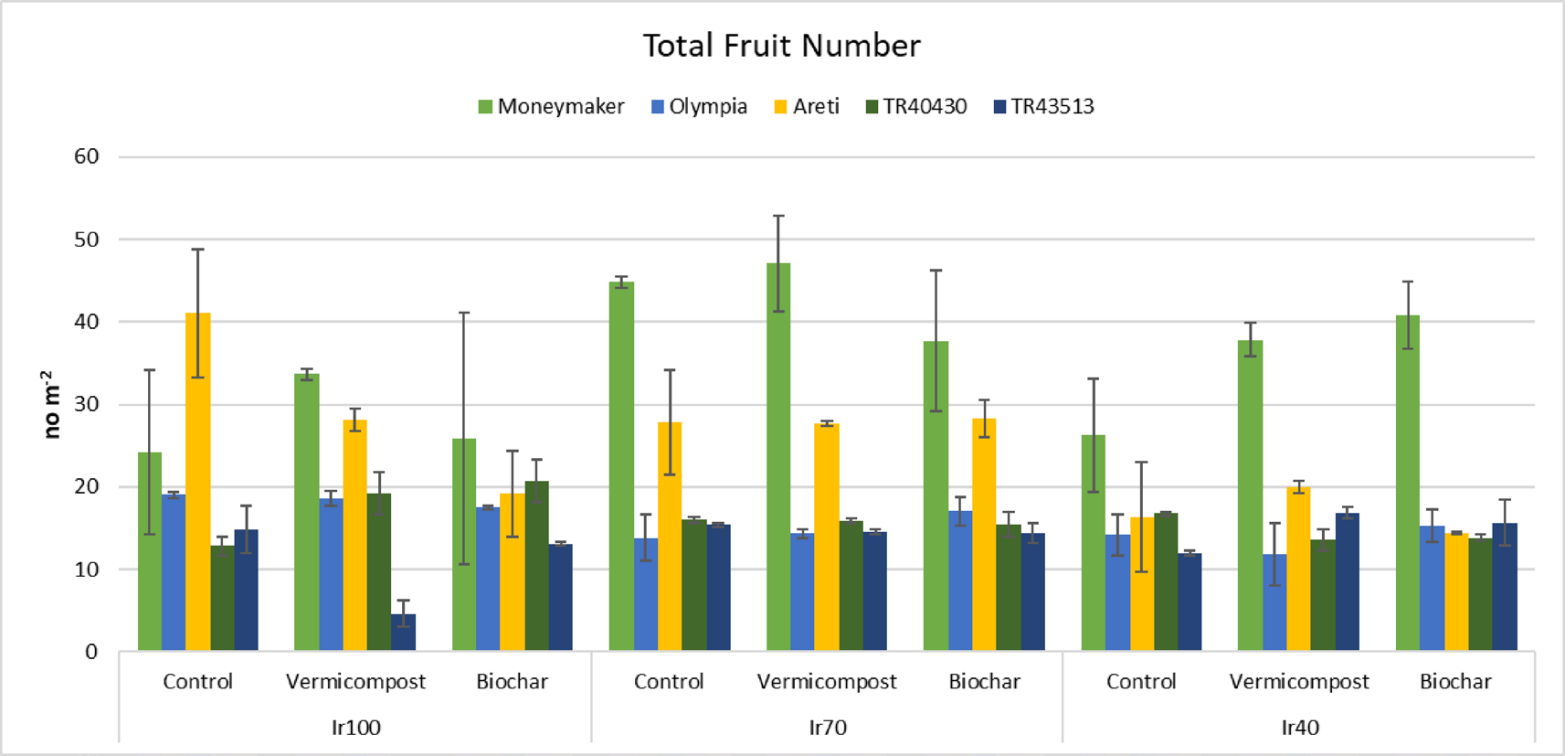
Interaction effects (*P*: 0.244) of irrigation, landraces, and organic amendments on total fruit number (Error bars show standard errors).

**Fig. 6:**
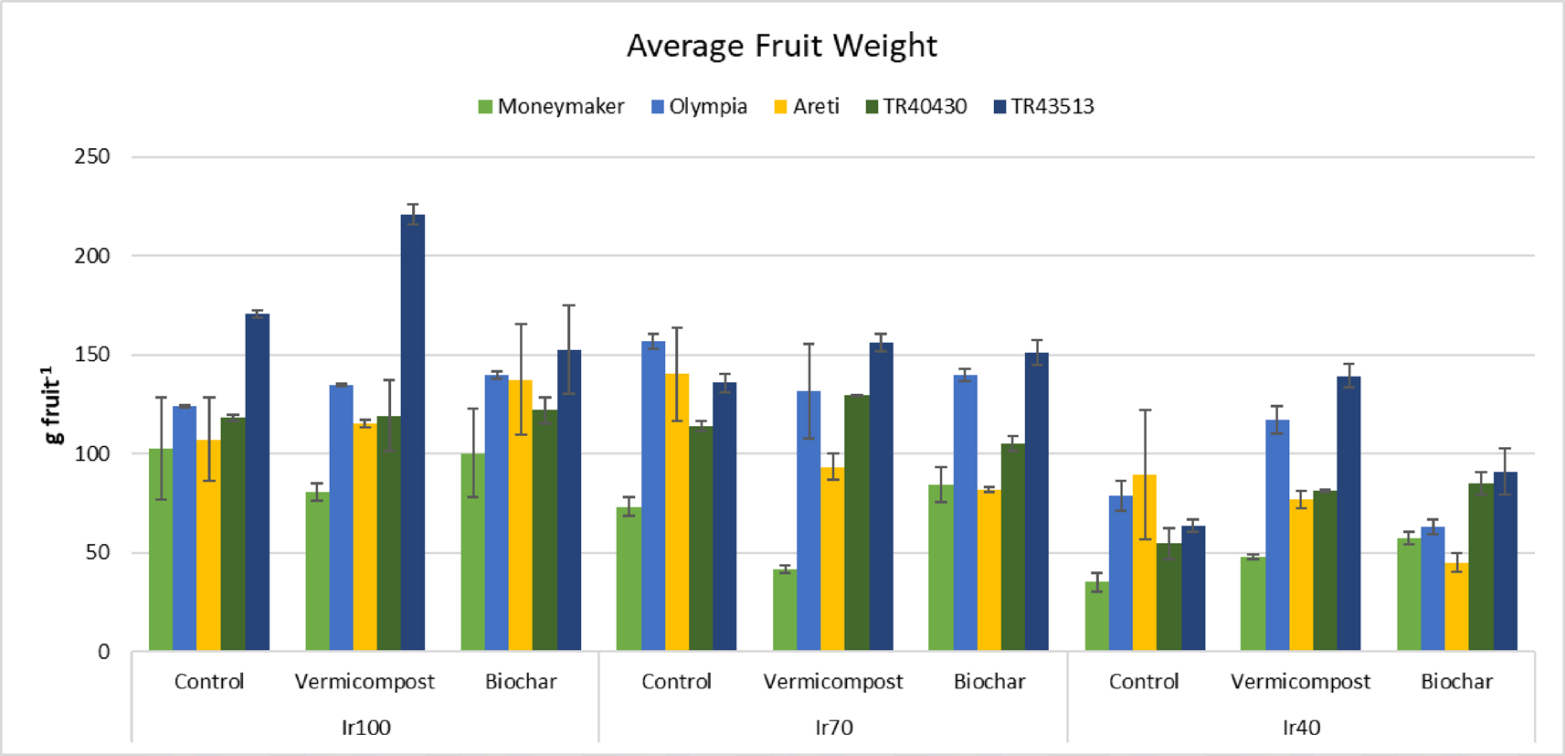
Interaction effects (*P*: 0.028) of irrigation, landraces, and organic amendments on average fruit weight (Error bars show standard errors).

The application of biochar and vermicompost positively influenced yield. The interaction of irrigation, landraces, and soil amendments was significant. Total yield ranged from 2.15 (Ir40 x Biochar x ‘Areti’) to 14.14 kg m^− 2^ (Ir100 x Control x ‘Areti’) (Fig. 4); total fruit number from 4.6 (Ir100 x Vermicompost x ‘TR43513’) to 47.1 no m^− 2^ (Ir70 x Vermicompost x ‘Moneymaker’) (Fig. 5) and average fruit weight from 35.2 (Ir40 x Control x ‘Moneymaker’) to 221.2 g fruit^− 1^ (Ir100 x Vermicompost x ‘TR43513’) (Fig. 6). Yield parameters decreased especially in Ir40; however, organic amendments mitigated the adverse effects.

### Water consumption and water use efficiency

**Fig. 7:**
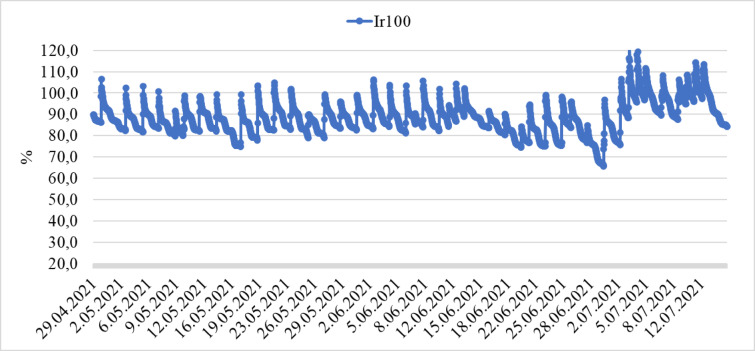
Changes in soil moisture under full irrigation.

**Fig. 8:**
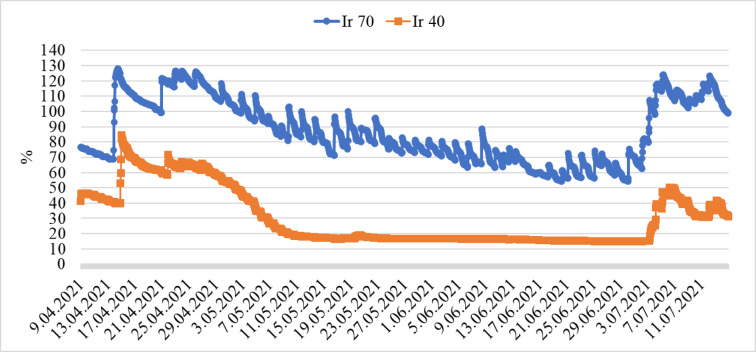
Changes in soil moisture under deficit irrigation Ir70 and Ir40.

During the growing season, soil moisture was monitored with sensors, and changes according to the irrigation levels are shown in Figs. 7 and 8. Irrigations were started without allowing the soil moisture capacity to fall below 80% for full irrigation. Irrigations were stopped when the soil moisture capacity reached 100%. In Ir70, the amount of applied irrigation water after the 2nd week remained below the field capacity, while in Ir40, soil moisture capacity remained below 70% two weeks after planting. The plants consumed the amount of irrigation water until it reached the moisture sensor.

The amount of irrigation water applied to the plants during the production period was 683.95, 456.74, and 302.27 mm for Ir100, Ir70, and Ir40, respectively. Irrigation applications were started three weeks after planting and monitored daily.

**Fig. 9:**
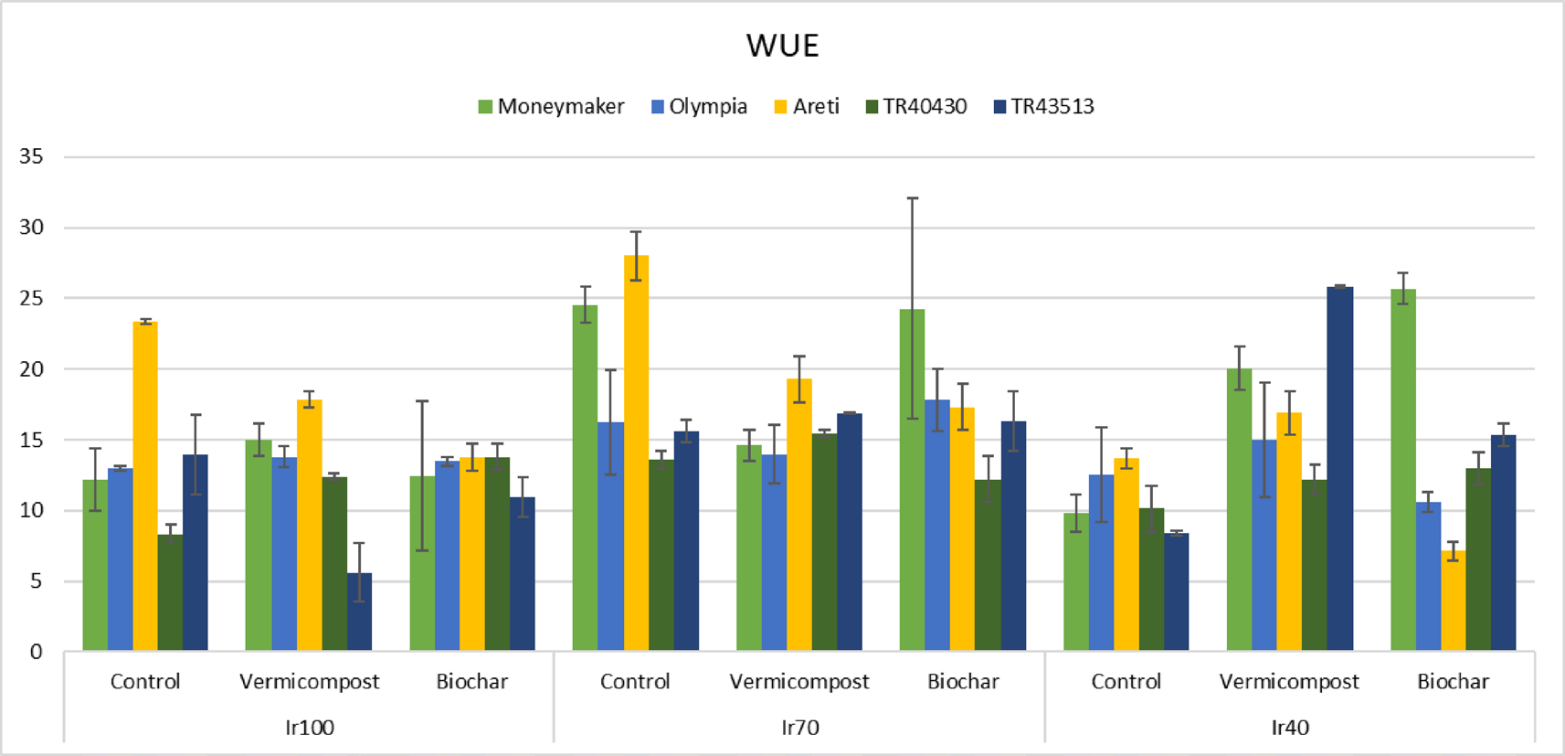
Interaction effects (*P*: 0.007) of irrigation, organic amendments, and landraces on Water Use Efficiency (Error bars show standard errors).

The main effects of irrigation treatments and landraces on WUE values were calculated based on total yield, and the interaction effects of treatments were found to be statistically significant. WUE was the highest in Ir70 and Moneymaker; the lowest WUE values were obtained from TR40430. In the same treatments, WUE values varied between 27.99 (Ir70 x CONT x ‘Areti’) and 5.62 kg m^− 3^ (Ir100 x VC x ‘TR43513’) (Fig. 9).

### Microbiological and enzymatic activities

**Fig. 10:**
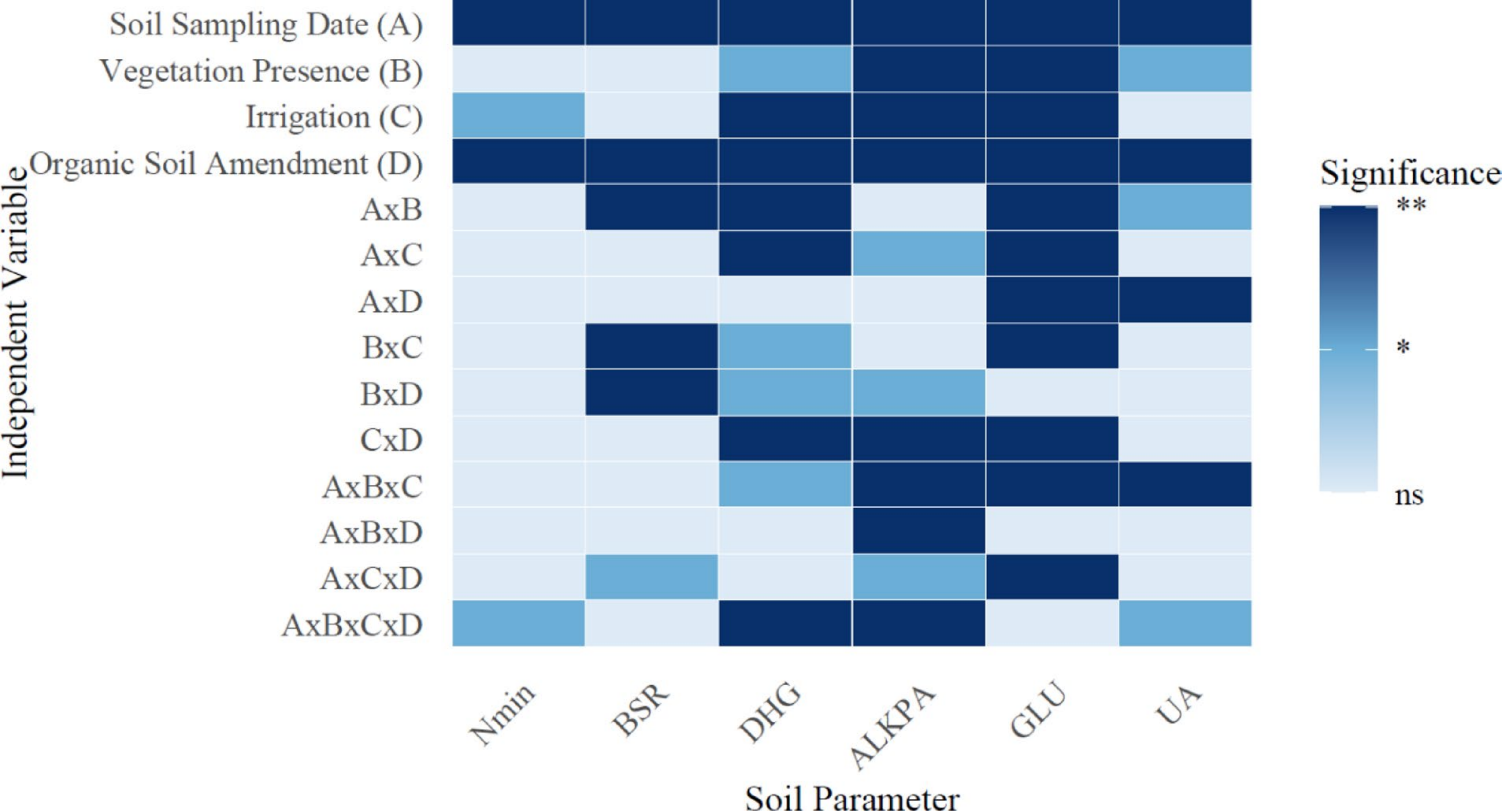
Main interaction effects of sampling date, vegetation presence, irrigation, and organic amendments on soil microbial properties (**: *P* < 0.01, *: *P* < 0.05, ns: not significant N_min_: potential N-mineralization (N_min_), BSR: basal soil respiration, ALKPA: alkaline phosphatase activity, DHG: dehydrogenase activity, GLU: β-glucosidase activity, UA: urease enzyme activity).

**Fig. 11:**
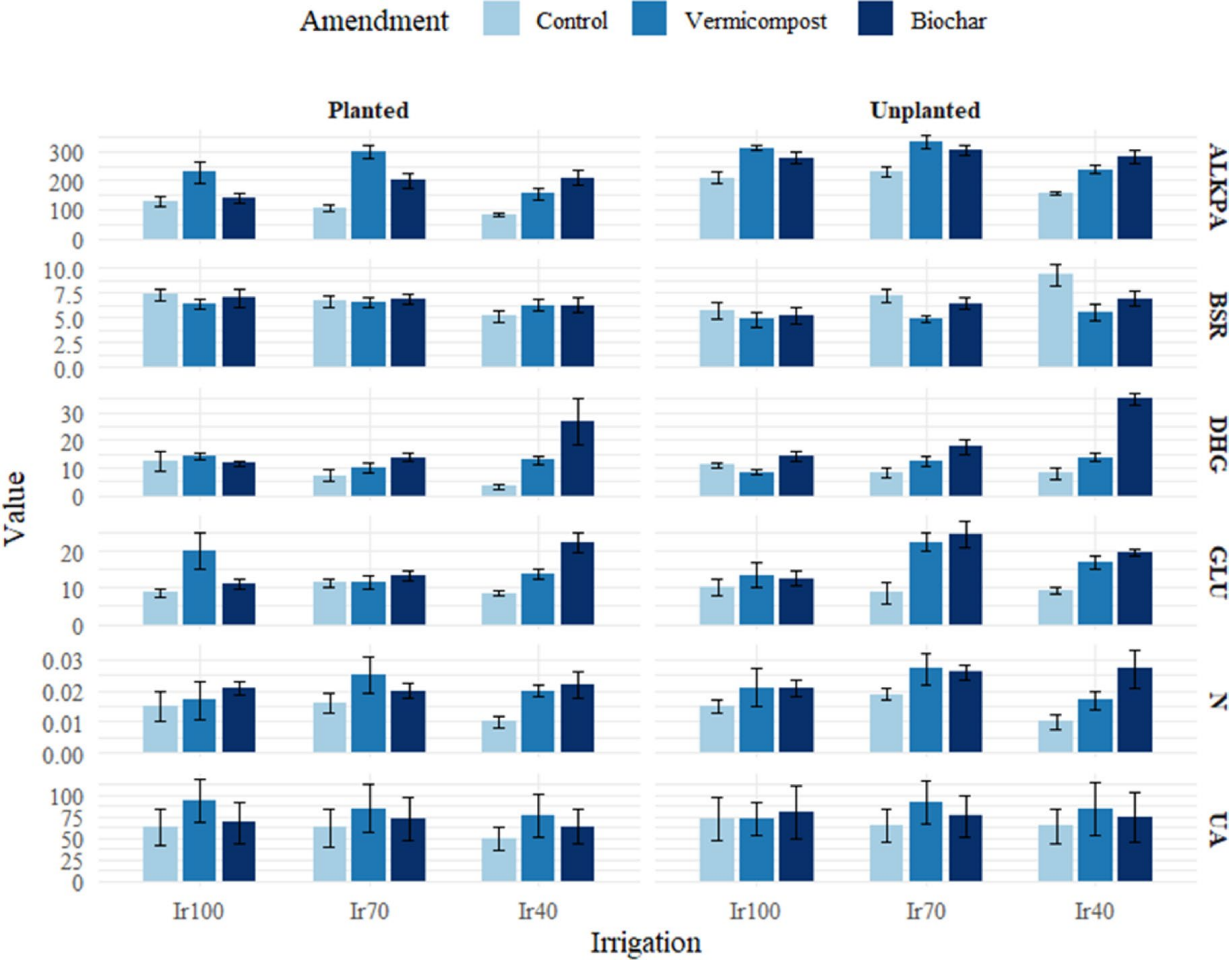
The average of microbial parameters for the growing cycle.

**Fig. 12:**
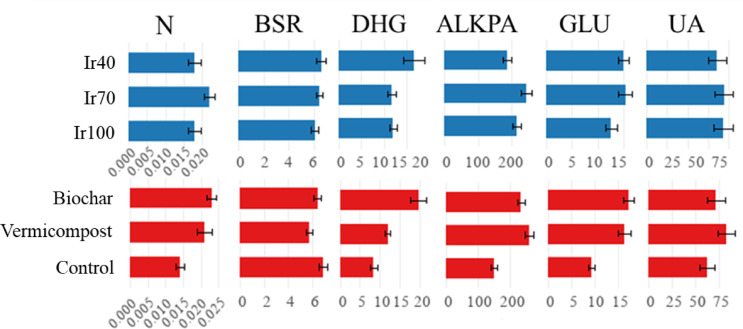
The main effects of treatments on soil microbial properties.

Figure 10 shows the significance level of soil sampling dates’ primary and interaction effects, vegetation presence, irrigation treatments, and organic amendments on soil microbial properties. Figures 11 and 12 present the average microbial parameters for the growing cycle and the main effects of treatments on soil microbial properties.

The application of biochar and vermicompost has shown a significant influence on potential nitrogen mineralization in soils. N_min_ changed between 0.004 and 0.35 µg NH_4_-N/g.h in the soil. Our study thoroughly examined the effect of organic amendments and irrigation regimes on basal soil respiration. Applying biochar significantly enhanced BSR, achieving an average rate of 6.43 µg CO₂-C/g soil.h, while control soils showed a BSR of 6.88 µg CO₂-C/g soil. h. These values were notably higher than the vermicompost soils, which had a BSR of 5.73 µg CO₂-C/g soil.h (*P* < 0.05). Dehydrogenase enzyme activity was significantly affected by the application of organic amendments and irrigation regimes in our study. Our results revealed that DHG activity was highest with biochar treatment, averaging 19.78 µg TPF/g soil. In contrast, vermicompost treatment exhibited a slightly lower but still significant activity of 12.06 µg TPF/g soil, compared to the control, which showed 8.46 µg TPF/g soil (*P* < 0.05). Vermicompost and biochar applications were observed to significantly elevate alkaline phosphatase activity compared to the control, reflecting the amendments’ role in enhancing microbial activity and nutrient cycling. Specifically, ALKPA activity under vermicompost treatment reached 260.39 µg PNP/g soil h. In contrast, biochar treatment resulted in 235.46 µg PNP/g soil.h, considerably higher than the control at 151.70 µg PNP/g soil.h (*P* < 0.05). β-glucosidase activity was significantly elevated using vermicompost, achieving 16.34 µg Saligenin/g 3 h. Biochar treatment resulted in 17.19 µg Saligenin/g 3 h, both higher than the control at 9.39 µg Saligenin/g 3 h (*P* < 0.05). The activity of the urease enzyme in the soil is significantly influenced by the application of organic amendments such as vermicompost and biochar, as well as different irrigation regimes. In our study, vermicompost application yielded the highest urease activity, measuring 83.48 µg N/g 2 h. This was followed by biochar application at 72.26 µg N/g.2 h, significantly higher than the control treatment, which showed 62.78 µg N/g.2 h (*P* < 0.05).

### Plant growth

The significance level of the primary and interaction effects of irrigation treatments, landraces, and organic soil amendments on measured morphological parameters is given in Table 5. At the end of the growing season, the main effects of irrigation, landraces, and organic amendment x landrace interaction on plant height were statistically significant. Plant height decreased with increasing water deficit. The plant height ranged from 0.67 (Ir40 x Vermicompost x ‘TR40430’) to 2.09 m (Ir100 x Vermicompost x ‘Moneymaker’) (Fig. S1, Supplementary material). When the Ir100 and Ir40 were compared, it was determined that the plant height decreased, excluding ‘Moneymaker’ and ‘TR43513’. Depending on the landraces, the decrease in control ranged from 7 to 27%, biochar from 2.2 to 24.5%, and vermicompost from 4.3 to 23%.

The main effects of irrigation treatments, organic amendments, and landraces on leaf thickness and the interaction of irrigation x organic soil amendment x landrace were statistically significant (Table 6). Leaf thickness increased with increasing water deficit and varied between 0.29 (Ir70 x Biochar x ‘Olympia’) and 0.31 mm (Ir70 x Vermicompost x ‘TR43513’) (Fig. S1, Supplementary material).

The fresh and dry weights of the vegetative part of the plant decreased with the increase in water deficit. The fresh weight of the vegetative part of plants ranged from 0.94 (Ir40 x Vermicompost x ‘TR40430’) to 3.31 kg plant^− 1^ (Ir100 x Vermicompost x ‘Olympia’), the dry weight ranged between 0.18 (Ir40 x Vermicompost x ‘TR40430’) and 0.56 kg plant^− 1^ (Ir100 x Control x ‘Areti’). Root fresh weight ranged from 0.22 (Ir100 x Biochar x ‘Areti’) to 0.08 kg plant^− 1^ (Ir100 x Vermicompost x ‘Moneymaker’), and dry weight from 0.02 to 0.05 kg per plant, and the highest dry weight was obtained from Ir40 x Control x ‘Areti’ (Fig. S1, Supplementary material).

**Table 5:** Main and interaction effects of irrigation, landraces, and organic amendments on plant morphological parameters.

Independent Variables	Plant Height	Leaf Thickness	Vegetative Part		Root	
			FW	DW	FW	DW
Irrigation (A)	**	**	**	**	**	*ns*
Landrace (B)	**	**	**	**	**	**
Org. Amendment (C)	*ns*	**	*ns*	*ns*	*ns*	*ns*
AxB	*ns*	**	*	*	**	*ns*
AxC	*ns*	*ns*	**	*	**	*ns*
BxC	**	*ns*	**	*ns*	**	*
AxBxC	*ns*	**	*ns*	*ns*	*ns*	*ns*

**: *P* < 0.01, *: *P* < 0.05, ns: not significant.

FW: Fresh weight, DW: Dry weight.

### Fruit quality

**Fig. 13:**
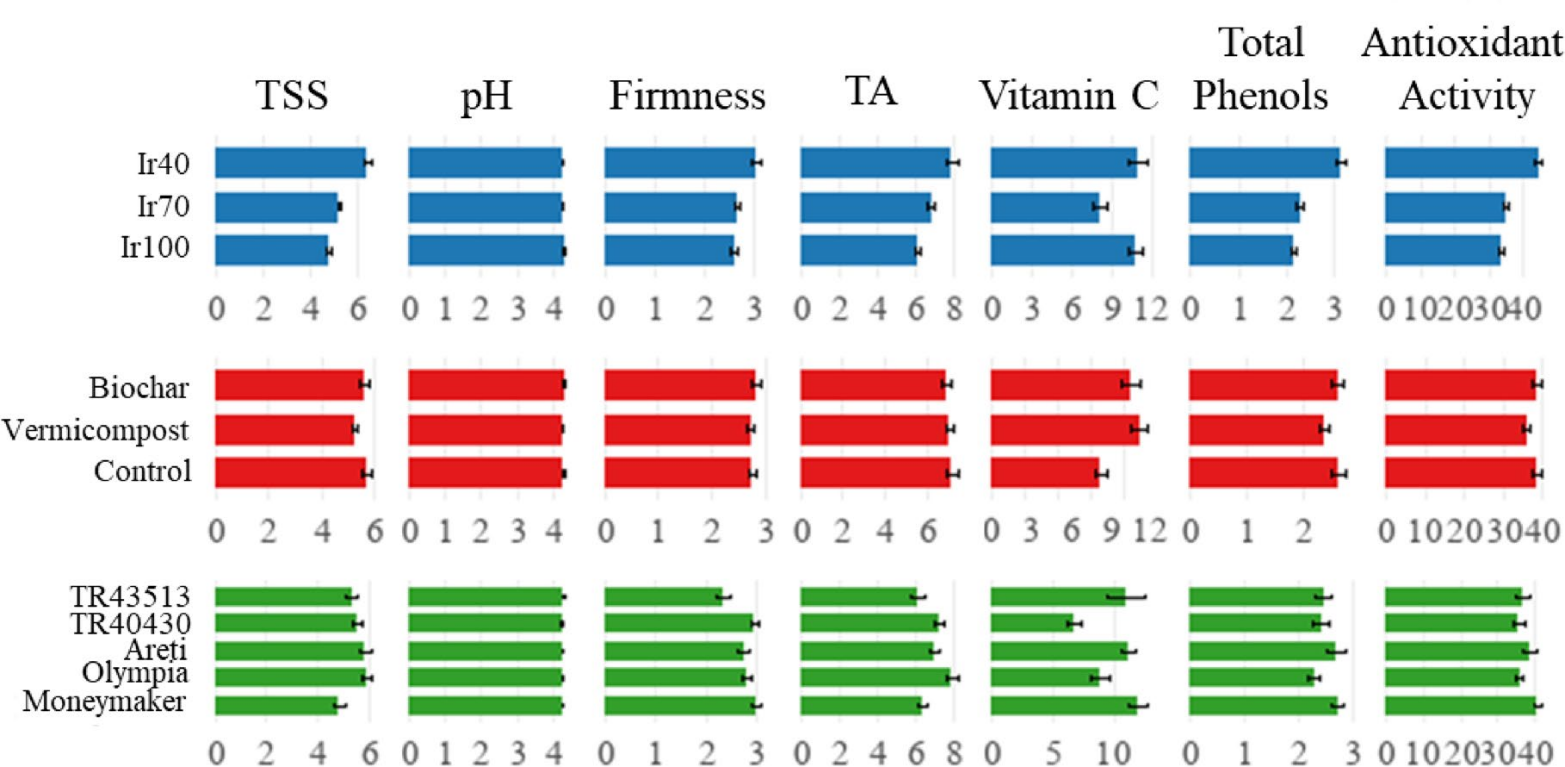
Main effects of irrigation, landraces, and organic amendments on fruit quality parameters.

The statistical effects of the treatments on the measured parameters are shown in Table 6, and the individual effects are shown in Fig. 13. The main and interaction effects of treatments on TSS and fruit juice pH were found to be significant. In Ir40, the average TSS was the highest and increased with the increase in water deficit, while the pH of fruit was the highest in Ir100. TSS ranged between 8.0% (Ir40 x Control x ‘TR40430’) and 3.73% (Ir100 x Vermicompost x ‘Moneymaker’). pH of fruit juice varied between 4.15 (Ir40 x Vermicompost x ‘TR40430’) and 4.39 (Ir100 x Vermicompost x ‘Olympia’). The main effects of irrigation treatments, landraces, and interactions, excluding landraces x organic amendments on fruit firmness, were found statistically significant. Fruit firmness varied between 2.02 (Ir70 x Control x ‘TR43513’) and 3.76 N (Ir40 x Biochar x ‘Areti’). TA was affected by the main effects of irrigation treatments and accessions and the interactions of all variables. The titratable acidity values changed between 5.04 (Ir70 x Control x ‘Moneymaker’) and 10.44 mval 100 mL^- 1^ (Ir40 x Biochar x ‘Olympia’) (Fig. S2, Supplementary material).

The effect of landraces on vitamin C content was significant, and the highest value was obtained from ‘Moneymaker’, while ‘TR40430’ had the lowest value. Vitamin C content varied between 20.10 (Ir40 x Biochar x ‘TR43513’) and 2.05 mg ascorbic acid 100 g^− 1^ (Ir70 x Biochar x ‘TR40430’). Although there were differences among landraces, an increasing trend was observed in vitamin C content with water deficit and organic amendment application. Total phenol content was higher in the Ir40 treatment, and it varied between 1.76 (Ir70 x Control x ‘Olympia’) and 4.60 mg GAE 100 g^− 1^ (Ir40 x Biochar x ‘Areti’). Antioxidant activity was also higher in Ir40, and it varied between 56.31 (Ir40 x Biochar x ‘Areti’) and 27.64 (Ir100 x Vermicompost x ‘TR40430’) µmol TE g^− 1^ (Fig. S2, Supplementary material).

**Table 6:** Main and interaction effects of irrigation, landraces, and organic amendments on fruit quality parameters.

Independent Variables	TSS (%)	pH	Firmness (Newton)	TA (mval 100 mL^− 1^)	Vitamin C (mg 100 g^− 1^)	Total phenols (mg GAE 100 g^− 1^)	Antioxidant activity (µmol TE g^− 1^)
Irrigation (A)	**	**	**	**	**	**	**
Landrace (B)	**	**	**	**	**	**	**
Org. Amendment (C)	**	**	*ns*	*ns*	**	**	**
AxB	**	**	**	**	**	**	**
AxC	**	**	**	**	**	**	**
BxC	**	**	*ns*	**	**	*	*ns*
AxBxC	**	**	**	**	**	*ns*	*

**: *P* < 0.01, *: *P* < 0.05, ns: not-significant, TSS: Total soluble solids, TA: Titratable acidity.

### Combined analysis of variance on total yield and water use efficiency

The study analyzed the main effects and interactions of irrigation, organic amendments, and landraces on total yield and water use efficiency (WUE) (Table 7). Significant differences were observed due to the combined influence of irrigation and organic amendment interactions (environment), landraces, and their interactions (GEI). These findings demonstrate that landrace performance is strongly influenced by irrigation levels and soil amendment treatments, underscoring the critical role of environmental conditions in determining yield potential and resource use efficiency.

**Table 7:** Additive main effects and multiplicative interaction (AMMI) analysis of variance for total yield and water use efficiency (WUE) of 5 landraces evaluated in 9 environments.

		Total Yield			WUE		
SOV	Df	SS	MS	%SS	SS	MS	%SS
Rep (Env)	9	11.43	1.27		40.21	4.47	
Env	8	304.87	38.11**		644.04**	80.51	
Gen	4	86.63	21.66**		383.15**	95.79	
Env x Gen	32	228.63	7.14**		1194.04**	37.31	
IPCA1	11	119.07	10.82**	52.1	647.34**	58.85	54.2
IPCA2	9	59.47	6.61**	26.0	291.47**	32.39	24.4
Residuals	36	74.97	2.08		352.78	9.80	
Total	121	935.17	7.73		3808.26	31.47	

**: significant at *P* < 0.01; SOV, Df, SS, MS indicate source of variation, degree of freedom, sum of squares, and mean sum of squares, respectively.

### Additive main effects and multiplicative interaction one biplot

**Fig. 14:**
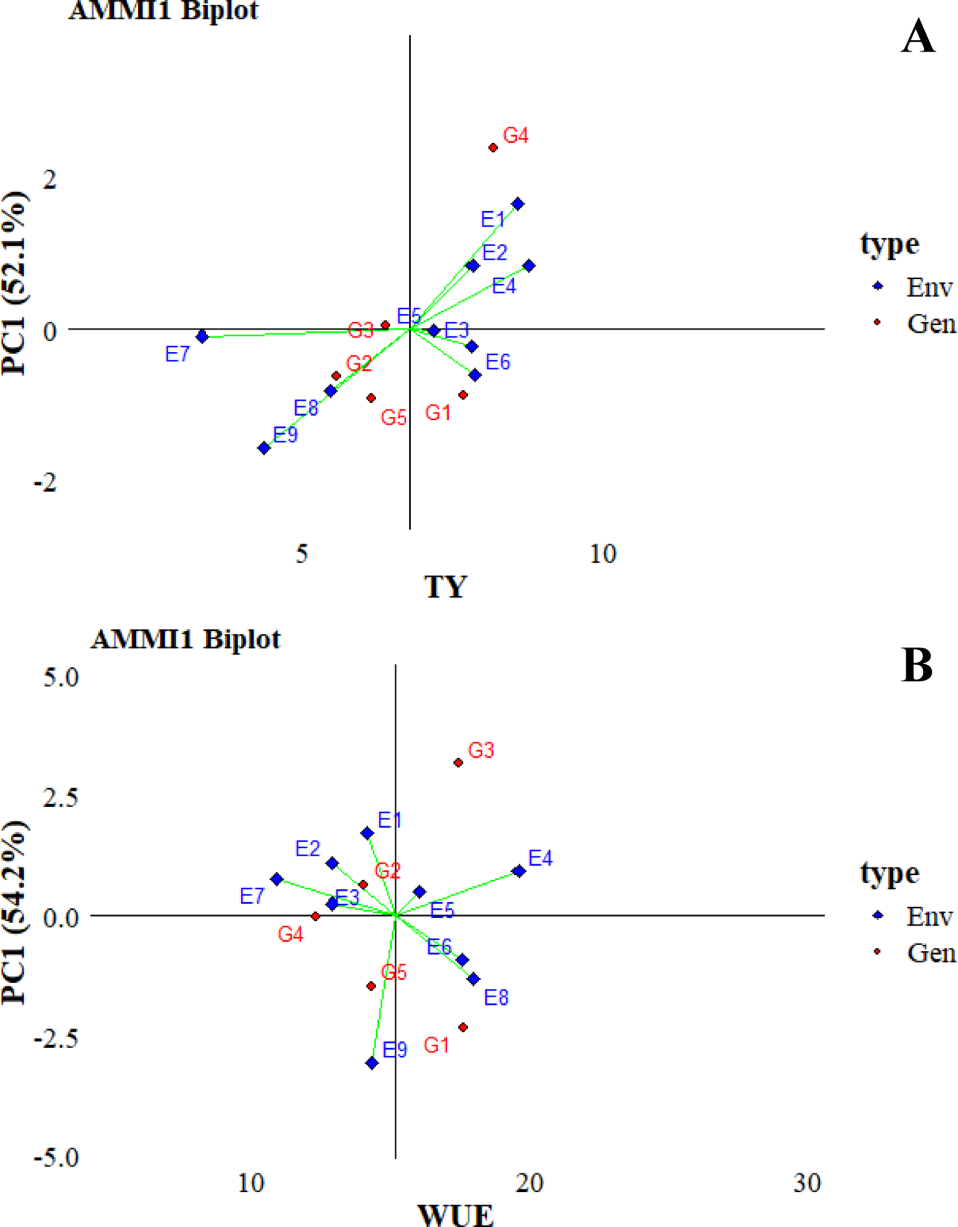
AMMI 1 biplot for total yield (**A**) and WUE (**B**).

The additive main effects and multiplicative interaction (AMMI) model is an appropriate statistical methodology to study genotype-environment interaction (GEI) and stability of individual genotypes and environments. In the AMMI 1 biplot, the graphical representation is realized in the axis and ordinate plane. These axes are associated with the first interaction principal component (IPCA1) term and the primary effects of total yield (Fig. 14A) and WUE (Fig. 14B) traits. IPCA1 scores were correlated with these variables in all contexts, providing helpful information on the genotype-environment interaction of the landraces studied. The analysis performed by GEI revealed both similarities and differences among the five landraces, as indicated by the IPCA1 values of 52.1% for total yield and 54.2% for WUE. The points for both environment and landraces were scattered in both graphs, indicating that the variability due to both effects was high, which is consistent with ANOVA. For total yield, landraces G1 and G4, and for WUE, landraces G1 and G3 were on the right side of the ordinate and gave higher values than other genotypes. For nine environmental conditions, landraces G3 in total yield, and G2 and G4 in WUE showed significant stability. The proximity of the data points to the origin and their near-zero values in PC1 indicate this. On the other hand, the landraces G4 for total yield and G3 for WUE showed promising results in the total mean, being to the right of the ordinate, but showed instability, moving significantly away from the axis. This showed a limited adaptive capacity for the traits available for these landraces, and environments characterized by limited conditions were found to be suitable.

### Additive main effects and multiplicative interaction two biplot

**Fig. 15:**
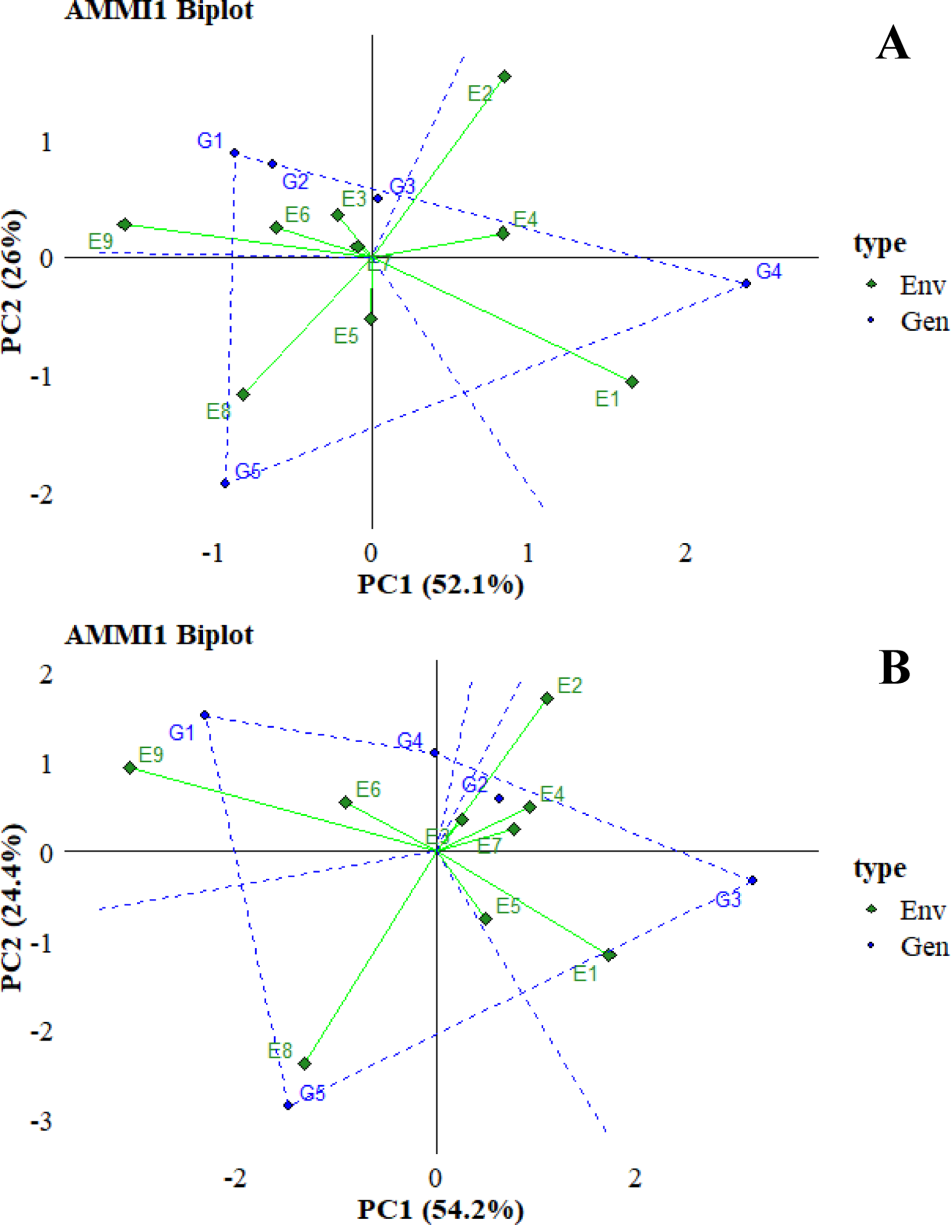
AMMI 2 biplot for total yield (**A**) and WUE (**B**).

AMMI 2 analysis supported the importance of including IPCA2 and IPCA1 scores to improve our understanding of genotype-environment interactions (GEI) in different environments. Furthermore, this methodology facilitated the identification of genotypic adaptations as shown in Fig. 15. The IPCA2 index values for two variables, total yield and WUE, were 26% and 24.4%, respectively. The results indicate that the first two principal components provide an adequate basis for projecting the AMMI model. The AMMI2 biplot uses distances from the biplot origin to provide information on the degree of interaction that genotypes exhibit between environments and vice versa. While environments close to the origin with low IPCA1 and IPCA2 scores can be said to have a significant impact on landrace stability, these environments exhibit a limited effect on genotype-environment interaction, indicating a wide range of adaptive environments for landraces. When the graphs were analyzed, in parallel with the AMMI1 graphs, G3 for total yield and G2 and G4 for WUE emerged as the landraces with the highest stability, as the points closest to the origin. When the lines of landraces and environments were analyzed, G5 gave good results in E8 for both traits. For total yield, G4 gave good results at E9, and for WUE, G1 gave good results at E9. This indicates that the organic amendments caused improvement in some landraces in other soil amendment treatments compared to the control treatments E4 and E7, which were more stable and gave lower values for all landraces.

When analyzed based on environments, for both total yield and WUE, E3 and E7 provided close values for all landraces as the nearest points to the origin and did not reveal any differences. Conversely, for both graphs, E2 and E9 emerged as the furthest points from the origin and were the distinguishing environments for the landraces.

## Discussion

Organic amendments represent a promising strategy for improving soil structure and promoting organic matter accumulation^73^. They can aid in restoring degraded soils, particularly in arid and semi-arid regions, while enhancing carbon sequestration, improving soil biodiversity, fertility, and supporting remediation efforts^74^. In Mediterranean soils, they contribute to carbon storage^75^ and increase bacterial populations under drought conditions^76^. In degraded Mediterranean soils, they can also improve chemical properties and bacterial diversity^77^. Moreover, organic amendments enhance plant growth and yield^78^In this study, biochar and vermicompost, combined with drought-tolerant landraces from various regions of the Mediterranean Basin, improved water use efficiency and positively affected yield, soil microbial activity, plant growth, and fruit quality under water stress conditions in the spring cycle at greenhouse conditions.

Our results showed that tolerant landraces reduced the amount of applied irrigation water by up to 30%. However, organic amendments are also considered an ideal choice to improve yield and plant growth in areas with low soil fertility and frequent stress waves, such as drought^79^. It was reported that biochar increased the net photosynthesis rate, promoted plant height and dry matter accumulation^80^improved nutritional value in cherry tomatoes^81^increased chlorophyll content in leaves, and positively affected yield^82^. Liu et al.^51^ investigated the effects of different doses of biochar and vermicompost on yield in greenhouse conditions and found that biochar and vermicompost increased tomato yield in doses. We observed that biochar and vermicompost applications increased the yield under water stress, and maintained physiological performance and microbial function. However, it varied depending on the landraces, having different fruit characteristics. Pistachio biochar and vermicompost also improved plant growth and yield in eggplant, particularly at 50% of plant water requirements^50^. This aligns with our observations that biochar enhances yield stability and physiological integrity under severe drought stress (Ir40). However, unlike this study, our results show that biochar also preserves or enhances soil enzymatic function, most notably urease and β-glucosidase activities, even under low moisture conditions. Thus, our study complements theirs by linking physiological performance and yield with biochemical indicators of soil health, providing a more holistic perspective on drought resilience strategies.

Water is crucial in plant production. However, climate change causes and worsens environmental stresses such as drought, heat, and salinity, which are strictly interconnected and lower the water available for crops. Therefore, any strategy minimizing water use and improving water use efficiency is critical^83^. In our study, the highest WUE values were obtained in Ir70^23^, and the effect of landraces was found to be significant^84^. The main impact of organic amendments was insignificant; however, the response showed differences in water deficit depending on landraces. Although soil conditioners do not significantly increase WUE, they indirectly increase water in the soil by changing soil structure and increasing water holding capacity^85^. The effect may be site-specific and depends on the origin of the amendment^86^.

Variation in soil moisture significantly impacts soil microbiota, affecting microbial biomass, declining enzymatic and disruption of nutrient cycling (e.g., carbon, nitrogen, and phosphorus)^87^all critical indicators of soil health^88,89^. This study demonstrated that biochar and vermicompost amendments substantially enhanced potentially mineralizable nitrogen under limited irrigation (Ir40) compared to control treatments. Biochar’s porous structure enhanced microbial colonization, while vermicompost’s nutrient-rich composition facilitated microbial enzyme activities^90,91^. These findings confirm earlier reports that organic amendments effectively promote nitrification and reduce nitrogen losses^92,93^. Moreover, basal soil respiration (BSR), an essential indicator of microbial metabolic activity, notably increased with biochar applications. However, literature indicates varying outcomes based on biochar properties and soil types^94,95^. Our results specifically highlight that both biochar and vermicompost reduced CO₂ emissions under water-deficit conditions due to their stable carbon content, emphasizing their role in mitigating soil carbon losses, despite some studies reporting increased microbial respiration^90,96^. Organic amendments also significantly influenced dehydrogenase activity, a key marker of microbial metabolic health^97^. Under deficit irrigation (Ir40), biochar exhibited the highest DHG activity, likely attributed to enhanced microbial habitat stability and moisture retention^98,99^. Although literature shows variability in biochar’s effectiveness^90,100^our study emphasizes its beneficial role in sustaining microbial activity under limited moisture conditions. Furthermore, alkaline phosphatase (ALKPA), vital for phosphorus cycling, was significantly enhanced by biochar and vermicompost applications, particularly under optimal moisture (Ir70), reinforcing the critical role of adequate moisture in enzyme function and microbial nutrient cycling^101,102^. Notably, biochar exhibited superior ALKPA performance under reduced irrigation (Ir40), reflecting the complexity of biochar-soil interactions and aligning with previously reported variability^103,104^. Additionally, vermicompost significantly elevated β-glucosidase (GLU) activity, essential for soil carbon cycling, confirming prior observations that organic amendments enhance enzymatic activities by providing microbial substrates^105,106^. Consistent with earlier studies^107^moisture levels critically influenced GLU activity, despite some conflicting reports on biochar’s role in enzyme stimulation^108,109^. Similarly, urease activity, fundamental in nitrogen cycling, peaked in vermicompost-treated soils due to favorable nutrient content and microbial stimulation^91,110^. Optimal moisture (full irrigation and Ir70) enhanced urease functionality, underscoring moisture’s importance in soil microbial dynamics and nutrient cycling^102,111^. Although biochar’s efficacy varied across studies^112,113^our results confirm vermicompost’s consistent effectiveness across different irrigation regimes^114^. Concerning plant growth, water deficit conditions reduced plant height and biomass but increased leaf thickness, indicating physiological stress adaptation. Organic amendments positively influenced plant growth, likely due to improved water retention, nutrient availability, and reduced oxidative stress. Our results align with studies reporting enhanced plant growth and nutrient uptake with biochar in various crops^115,116^and similarly, vermicompost positively affected growth in eggplant and tomato^117,118^. Our data contribute to the nutrient uptake by providing enzymatic evidence, such as increased phosphatase and urease activities, showing that these materials also support microbial nutrient cycling under water-deficient conditions. This confirms that the physiological resilience conferred by bio-organic amendments is rooted in improved water relations and sustained soil biological functioning under stress. Overall, this study reinforces the positive role of vermicompost and biochar amendments in enhancing microbial activity, enzymatic functions, and plant growth under varying irrigation conditions, while also highlighting the inherent variability observed across studies, indicating the necessity of context-specific research to optimize these amendments for sustainable agricultural management.

Regarding plant growth, plant height and fresh and dry weights of vegetative parts decreased, and leaf thickness increased with water deficit. Studies with different species revealed that biochar increased plant height and yield in wheat^115^decreased cadmium and sodium uptake in rice^119^and increased nutrient element content of cabbage seedlings^116^. Vermicompost increased plant height in eggplants^117^ and tomatoes^118^. In our study, the positive effect of organic amendments on plant growth was observed depending on landraces^10^which could be attributed to the improvement of soil water retention and water holding capacity and reduction of osmotic and oxidative stressors^43^.

Biochar has been reported to enhance soil structure, water retention, and nutrient availability, promoting better root development and plant growth^95^. The ability of biochar to retain soil water depends on the combination of its porosity and the chemical functionalities present on its surface^120^ resulting in enhancement of the physico-chemical properties of amended soils^121^. Vermicompost, rich in essential nutrients and microbial activity, further supports plant health by improving soil fertility and microbial dynamics^91^. Vermicompost also contributes significantly to pH stabilization^122,123^. Our findings are consistent with previous studies demonstrating improved biomass and yield in crops treated with biochar and vermicompost^124^. Water stress/deficit adversely affects plant growth and productivity^125^ and the response of tomato crop varies according to the duration of stress, developmental stage^126,127^ and genotype^128–132^.

Irrigation deficit may favorably affect fruit quality parameters of tomato crops. For instance, water deficit has improved antioxidant activity^133,134^ total soluble solids, acidity^135^dry weight^136^fruit firmness, organic acids (sucrose and citric acid), active metabolic compounds such as vitamin C^127^ and reducing sugars^132^and vitamin C and carotenoid content^137^. Dere et al.^138^ compared nine drought-tolerant genotypes and two commercial varieties, and they found that vitamin C content, total phenols, flavonoids, antioxidants such as SCPC, and β-carotene increased under drought conditions. In contrast, the response of genotypes showed differences. Those results are in harmony with ours.

The AMMI (Additive Main Effects and Multiplicative Interaction) model is a hybrid analysis that merges analysis of variance (ANOVA) with principal component analysis (PCA) into a cohesive approach^139^. It is frequently used in multi-environmental trial analysis^140^. When used to examine genotype–environment interactions—such as those involving irrigation and organic amendments—the AMMI model reveals that environmental effects on tomato varieties vary, with landraces responding differently regarding yield and related traits. Similar patterns have been extensively documented in studies of other crops^141–144^.

The ANOVA test showed that the effect of the genotype-environment interaction was divided into two IPCAs, indicating the magnitude of the impact and that the genotypes tested responded differently under different environmental conditions^145^. It has been shown that when the first two components of the interaction (IPCA) explain most of the variation in landrace traits, it is sufficient to identify better landraces^146,147^. In addition, Habtegebriel^148^ showed that IPCA1 genotype scores can help assess yield stability. Furthermore, AMMI-1 and AMMI-2 biplots evaluated the overall adaptability of the studied genotypes. When the results are analyzed, G4 for total yield, although not stable, gave the best result in the general average, but showed the lowest results in terms of WUE. For WUE, G1 and G3 gave good results, and G1 was more stable than G3. In terms of being affected by environmental conditions, although they did not provide the best results, it was observed that G3 for total yield and G2 for WUE maintained their value stability.

## Conclusion

Water scarcity, exacerbated by global climate change, presents a challenge impacting all sectors, particularly agriculture. Our research under controlled greenhouse conditions demonstrated that applying stable carbon-rich organic amendments, such as vermicompost and biochar at 10 t ha^− 1^, facilitated the negative impacts of limited irrigation on plant growth, yield, water use, and soil microbial activity. Specifically, irrigation at Ir70 levels, supplemented with vermicompost or biochar, significantly enhances microbial parameters. Moreover, these amendments maintained or improved microbial activity under the more restrictive Ir40 irrigation regime. Vermicompost and biochar differentially supported microbial activity depending on the irrigation level, with vermicompost being more effective under moderate water restriction (Ir70) and biochar showing benefits under severe restriction (Ir40). The higher microbial activity observed in untreated soils underscores the detrimental impact of physical soil disturbance and chemical treatments on soil microorganisms. However, it should be noted that these findings vary depending on the landraces. Among the tested landraces, Areti was found comparable to the control cv. ‘Moneymaker’ in terms of water use efficiency. These findings highlight the critical role of organic amendments in enhancing soil resilience to water scarcity, thereby contributing to sustainable agricultural practices in the face of climatic challenges.

Although the experiment was conducted in greenhouse conditions for controlled drought simulation, the results provide transferable insights for open-field tomato systems where water scarcity poses a significant challenge. Given the increasing adoption of protected cultivation practices in Mediterranean agriculture, the microbial and agronomic responses observed here can inform scalable drought-resilient strategies. The use of organic amendments can enhance economic viability by improving soil fertility and increasing crop productivity. Over time, this can lead to increased profitability, reduced environmental impact, and greater long-term sustainability for agricultural system.

## Electronic supplementary material

Below is the link to the electronic supplementary material.

Supplementary Material 1

## Appendix. Supplementary materials

Supplementary material associated with this article is provided in the supplementary file.

## Data Availability

Data will be made available from the corresponding author upon request.

## Abbreviations

ALKPA: Alkaline phosphatase activity
AMMI: Additive main effects and multiplicative interaction
BIO: Biochar
BSR: Basal soil respiration
CONT: Control
Df: Degree of freedom
DHG: Dehydrogenase activity
DW: Dry weight
FW: Fresh weight
GEI: Genotype-environment interaction
GLU: β-glucosidase activity
HCl: Hydrochloric acid
IPCA: Interaction principal component axis
Ir: Irrigation
MS: Mean sum of square
Nmin: Potential N-mineralization
SS: Sum of square
SOV: Source of variation
TA: Titratable acidity
TSS: Total solube solids
UA: Urease enzyme activity
WUE: Water use efficiency
VC: Vermicompost
