# Supplementary material for: Enhancing tomato drought resilience with organic amendments and local landraces
Source: Sci Rep. 2025 Jul 18;15:26172. doi: 10.1038/s41598-025-12098-0 (PMC12274364; doi:10.1038/s41598-025-12098-0)
Supplement: Supplementary file 1 — Supplementary Material 1 [file 41598_2025_12098_MOESM1_ESM.docx]

**Supplementary materials**

**Figure S1.**

Triple interaction effects of treatments on plant height, leaf thickness, fresh and dry weights of vegetative part and roots.

**
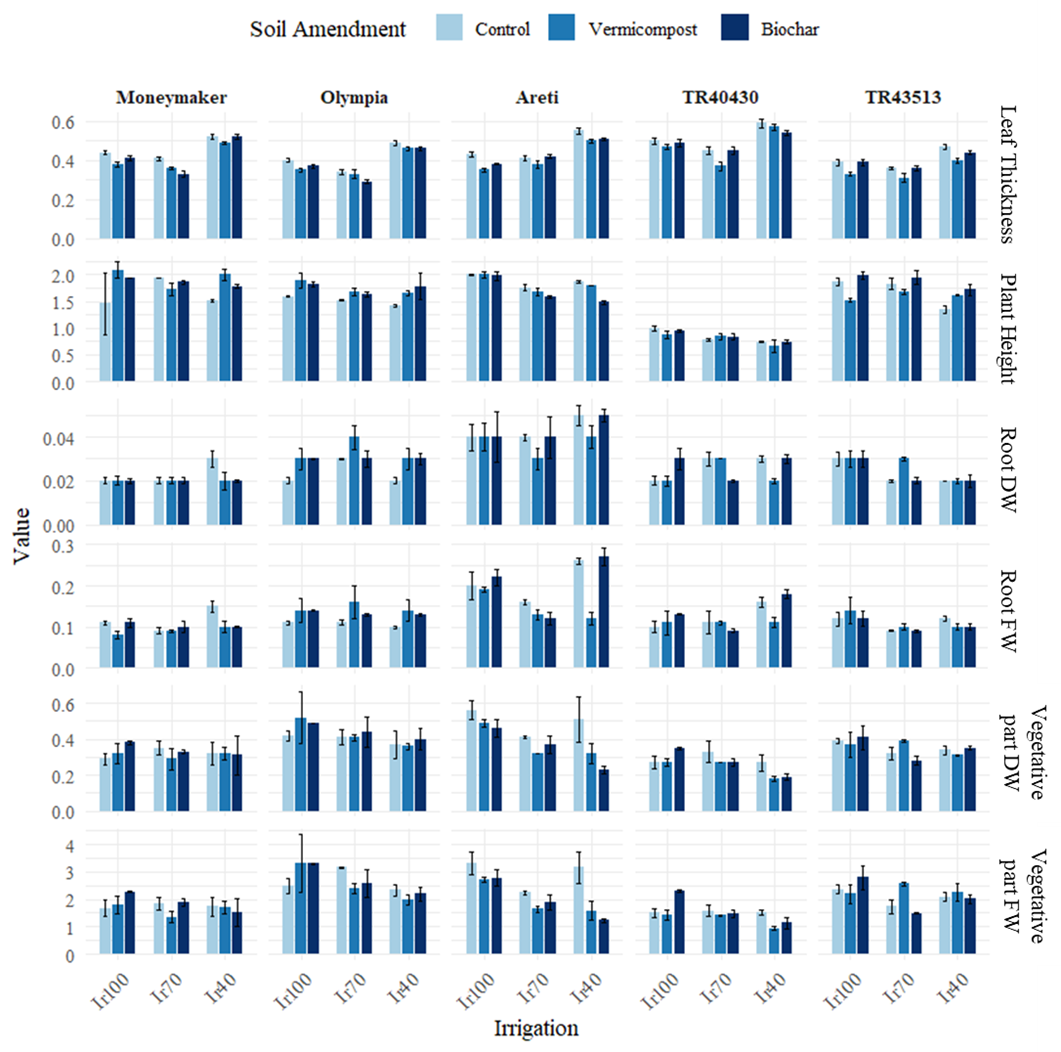
**

**Figure S2.**

Interaction effects of treatments on fruit quality paramete


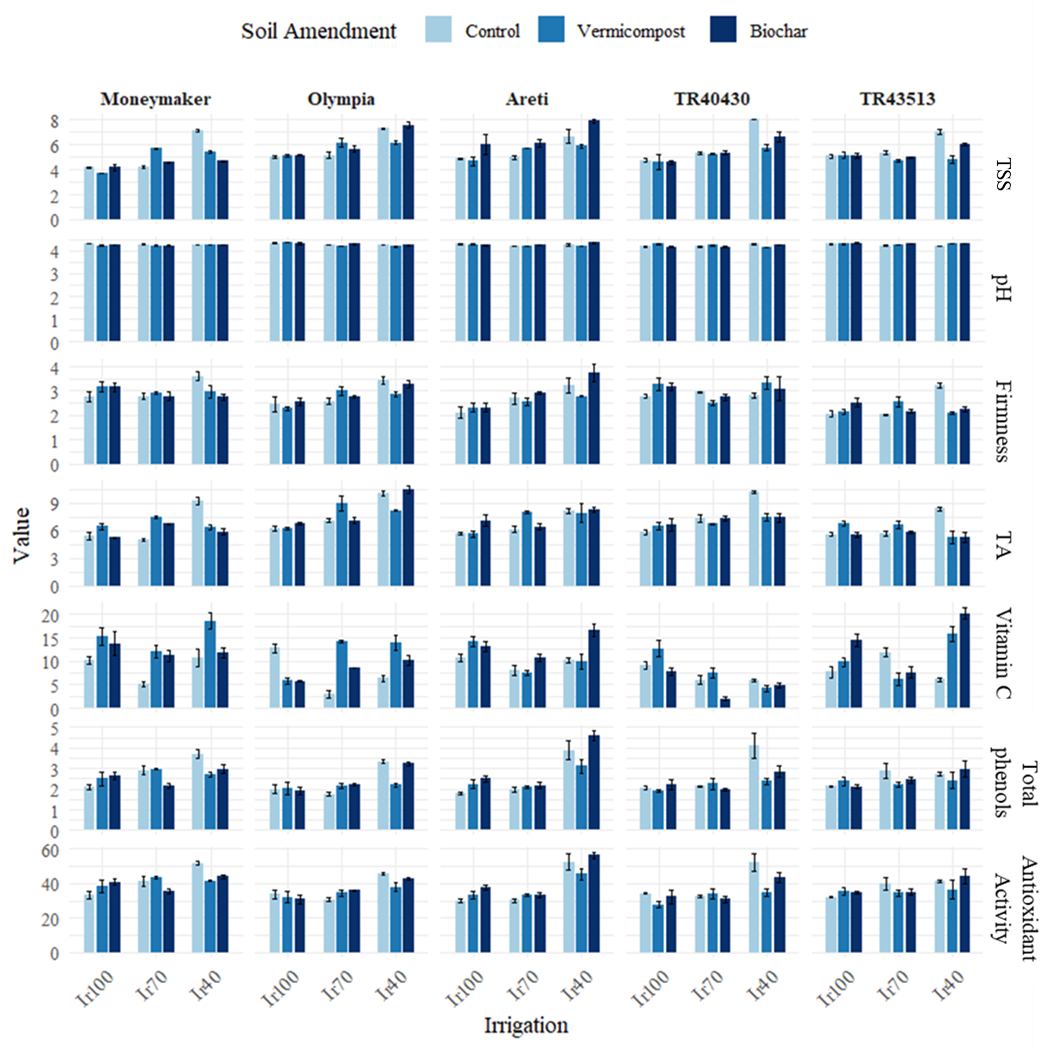
TSS: Total soluble solids, TA: Titratable acidity,
